# Assessment of a Business-to-Consumer (B2C) model for Telemonitoring patients with Chronic Heart Failure (CHF)

**DOI:** 10.1186/s12911-017-0541-2

**Published:** 2017-10-11

**Authors:** Andrija S. Grustam, Hubertus J. M. Vrijhoef, Ron Koymans, Philipp Hukal, Johan L. Severens

**Affiliations:** 1 Erasmus School of Health Policy & Management, Erasmus University Rotterdam, Rotterdam, Netherlands; 2 Professional Healthcare Services & Solutions, Philips Research, Eindhoven, Netherlands; 3 Department of Patient & Care, Maastricht UMC, Maastricht, Netherlands; 4 Department of Family Medicine and Chronic Care, Vrije Universiteit Brussels, Brussels, Belgium; 5 Panaxea b.v, Amsterdam, Netherlands; 6 Information Systems and Management, Warwick Business School, Coventry, UK; 7 iMTA, Institute of Medical Technology Assessment, Erasmus University Rotterdam, Rotterdam, Netherlands

**Keywords:** Business model, Financial analysis, B2C, Telemonitoring, CHF

## Abstract

**Background:**

The purpose of this study is to assess the Business-to-Consumer (B2C) model for telemonitoring patients with Chronic Heart Failure (CHF) by analysing the value it creates, both for organizations or ventures that provide telemonitoring services based on it, and for society.

**Methods:**

The business model assessment was based on the following categories: caveats, venture type, six-factor alignment, strategic market assessment, financial viability, valuation analysis, sustainability, societal impact, and technology assessment. The venture valuation was performed for three jurisdictions (countries) – Singapore, the Netherlands and the United States – in order to show the opportunities in a small, medium-sized, and large country (i.e. population).

**Results:**

The business model assessment revealed that B2C telemonitoring is viable and profitable in the Innovating in Healthcare Framework. Analysis of the ecosystem revealed an average-to-excellent fit with the six factors. The structure and financing fit was *average*, public policy and technology alignment was *good*, while consumer alignment and accountability fit was deemed *excellent*. The financial prognosis revealed that the venture is viable and profitable in Singapore and the Netherlands but not in the United States due to relatively high salary inputs.

**Conclusions:**

The B2C model in telemonitoring CHF potentially creates value for patients, shareholders of the service provider, and society. However, the validity of the results could be improved, for instance by using a peer-reviewed framework, a systematic literature search, case-based cost/efficiency inputs, and varied scenario inputs.

**Electronic supplementary material:**

The online version of this article (10.1186/s12911-017-0541-2) contains supplementary material, which is available to authorized users.

## Background

Populations globally are aging, chronic diseases are becoming more prevalent and healthcare budgets are strained. Telehealth, i.e. telecommunication technologies used in healthcare, are emerging rapidly to help cope with the ever-increasing number of people suffering from chronic diseases. In the current healthcare climate, where a quarter of countries worldwide have a telehealth policy in place [1], the dominant financial strategy is based on reimbursement schemes. This is also referred to as the Business-To-Business model (B2B). However, there are many barriers to the uptake of telehealth under the B2B model [2].

In a previous publication we designed a Business-to-Consumer (B2C) model for telemonitoring patients with Chronic Heart Failure (CHF), by extending the existing B2B model. In order for CHF patients to have access to this service, healthcare providers, equipment manufacturers, regulators/payers, and promoters/distributors must come together via the establishment of a telemonitoring centre in a jurisdiction. The B2B model needs to be extended toward the B2C to create synergies between these players in the healthcare ecosystem. However, it is not known if this model creates value for the proposed venture and society.

**Fig. 1:**
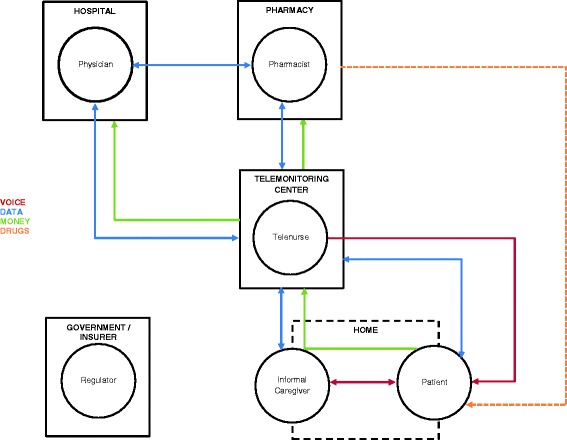
Individual and institutional communication in the B2C model for telemonitoring patients with chronic heart failure. Telenurse occupies the central role in the B2C telemonitoring model, and coordinates the care between the physician, pharmacist, patient, and informal caregiver, from the telemonitoring centre. The flow of voice communication (red line), data (blue line), reimbursement (green line), and drugs (orange line) can be unidirectional or bidirectional. It is represented by arrows between the agents or their respective institutions. The B2C telemonitoring decouples the patient and the informal caregiver from receiving care in the home. The payer in the B2C model is the patient, thus the government plays a role of the regulator only

The venture is based on patient-driven demand for telemonitoring of cardio-vascular disease in the future. The targeted customer is: 1) a person aged 55+ at risk of or suffering from CHF, 2) with smartphone and mobile internet, and 3) able to procure the service and the telemonitoring devices. Care coordination is performed by telemonitoring nurses based in a telemonitoring centre. A physician, pharmacist, and informal caregiver are included in the care process, and each stakeholder can set up and invite other stakeholders to join the care-coordination team. The patient is still a part of the healthcare system, which pays for the physician time and service, but is able to receive telemonitoring service irrespective of the space-time restrictions (e.g. on the road, during the weekend). The procurement of the drugs, and the reimbursement for healthcare system utilization, goes via the regular pharmacies and insurance companies/ national health systems. Figure 1 describes the flow of data, voice communication, money, and medication between the stakeholders.

**Fig. 2:**
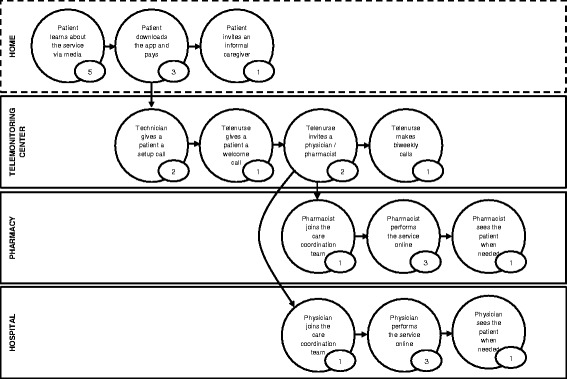
Care Experience Flow Map of the B2C model for telemonitoring patients with chronic heart failure (time in minutes). The map shows the experience of the patient on the B2C telemonitoring service, with a flow from one state to another, and time in minutes spent in each state. The flow is segmented according to the institution of care. The map shows what the experience of exchange in the B2C telemonitoring is, and is not exhaustive. *Adapted from Patient and Family Centered Care Innovation Center of UPMC (PFCC, 2016)*

From an execution perspective, B2C telemonitoring involves: 1) informing patients via the media that the telemonitoring service exists, 2) patients and other stakeholders downloading the app, 3) patients registering and paying the installation charge and monthly fee, 4) connection of the peripheral monitoring devices, 5) technical assistance to resolve any installation issues, and 6) a telemonitoring nurse making an initial call to the patient. Figure 2 presents the Care Experience Flow Map [3] with the estimated number of minutes each action requires.

The aim of this paper is to assess the B2C model in telemonitoring patients with CHF by analysing the potential value it creates for 1) organizations or ventures that provide telemonitoring services based on this model, and 2) society. Our hypothesis is that B2C telemonitoring is well aligned with current healthcare structures, financing possibilities, public policy, technology availability, and consumer demands.

## Methods

We used a non-peer-reviewed method “Innovating in Healthcare Framework” to assess the B2C model for telemonitoring CHF [4]. We searched the literature in order to inform both the non-financial part of the assessment and the financial calculations (i.e. the MS Excel model creation). We took a critical look at our assessment by discussing caveats and limitations.

### Literature search

Both peer-reviewed and non-reviewed sources were taken into account. Scientific literature in English from 2010 onwards was searched. NHS Evidence, TRIP Database, Cohrane Library, PubMed, Medline, EMBASE, CINAHL, Web of Science, and Scopus were searched for “telemonitoring”, “costs”, “financial”, “CHF”, and combinations thereof. Business literature, such as Harvard Business Review, and reports from respected consulting firms were searched for the same terms. Our intention was not to do the systematic literature search, but to inform the assessment. We opted for a convenience approach, selecting literature based on ease of availability [5], and did not use exclusion criteria. AG performed the search and the selection.

### The framework

The Innovating in Healthcare Framework [4] is divided into three parts, consisting of ten main elements: 1) caveats – “Life is not literature”, “Basic is beautiful”, and “Beware of true believers”, 2) venture type, 3) six-factor alignment – structure, financing, public policy, technology, consumers, and accountability, 4) strategic market assessment, 5) financial viability – breakeven and market share analysis, 6) valuation analysis, 7) sustainability – revenues, costs, management, and technology, 8) managerial assessment (not performed due to early stages of venture ideation), 9) societal impact, and 10) technology assessment. See Additional file 1 for detailed framework structure.

#### The three distinctly different types of healthcare innovation

Healthcare ventures can be consumer-focused, technology-based, or integrators [4]. Consumer-focused ventures involve patients/consumers in their own care. Technology-based ventures rely on scientific advances to provide new treatments and cures, while the integrators rely on “horizontal and/or vertical integration to achieve healthcare efficiency and quality benefits” [4]. Understanding the type is important for assessing the business model, how it fits with the six factors, and which cost items should go into the financial analysis.

#### Six-factor alignment: Is the idea viable?

The six factors that most critically influence innovation in healthcare are: 1) structure, 2) the nature of financing, 3) public policy, 4) technology, 5) consumers, and 6) accountability [6]. Structure deals with the established organizations and their power dynamics in the healthcare market. Financing considers reimbursement policies and available sources of capital. Public policy promotes activities in a society, or creates distortions in the market which can work against innovation. Technology is necessary in order to provide new ways of treatment, but is by no means sufficient. Consumers are the players in the market, usually time stressed, not empowered or satisfied with the care offering. Finally, accountability is key to the long-term success of a venture, be it toward the customers or the shareholders. It is imperative to understand the interconnections between these components in order to succeed in the healthcare market.

AG, RK and BV graded the B2C model according to its fit with the six factors. No blinded scoring was performed, nor inter-rater variation assessed. We used a 4-point Likert-type scale [7] to assess the fit by consensus. The scale ranges from *non-existent, poor,* and *good* to *excellent.*

#### Business model elements: How to make it happen

In the third part of the assessment we analysed market size, competitive strategy used, and the venture’s financial viability. The calculations were performed in 2016 US dollars. Personnel costs were converted from Singaporean dollars (SG) and euros (NL) to US dollars (July 2016). Non-personnel costs and volumes were expressed in US dollars and assumed to be equal in all three countries.

The breakeven analysis considered the number of patients needed for profits to surpass costs. If the venture is not able to reach the breakeven point (easily), it is not viable. Breakeven analysis is an important tool for profit planning [8]. It is also referred to as “cost-volume profit analysis”, and describes the volume of patients needed for the service to generate a profit.

The final step of the analysis involves assessing venture sustainability, managerial aspects (not performed in this publication due to early stage ideation phase), societal impact and technology-related risks [4].

### Financial calculations

The venture valuation was performed for legal jurisdictions, i.e. countries – Singapore, the Netherlands and the United States – in order to portray the opportunity in a small, medium-sized, and large country (i.e. population). These countries were chosen because smartphone penetration there is among the highest in the world – Singapore 88%, The Netherlands 76%, and the US 57% [9]. Also, the state of telehealth in these countries is among the most advanced, and thus favourable for implementation of telemonitoring technology via the new business model. Singapore‘s healthcare system ranks among the best and most efficient in the world, e.g. 2nd for healthcare outcomes by The Economist Intelligence Unit Healthcare (2014). The same report puts The Netherlands in 6th place for spending on healthcare but 17th for outcomes, while the United States is first for spending but 32nd for health outcomes [10]. The financial model was created in MS Excel 2013, with 5-year projections based on the historical data retrieved by the literature searches.

The fictitious B2C telemonitoring venture provides a 10-min consultation with a telenurse every fortnight, which is similar to the Centre for Telehealth, University of Hull, where one telenurse provides a 15–20-min consultation once per month. Advanced telehealth systems can support more than 250 patients per nurse [11], but we conservatively based our calculations on one telenurse taking care of 200 patients. The technician makes a 20-min call once a year (at set-up and for yearly maintenance), and is able to service 250 patients per month. There is one manager per 30 staff members. Holidays, sick leave, maternity leave, and personnel churn is accounted for as spare capacity of 15%.

Cosentino acknowledges that “determining clinical staffing requirements and nurse-to-telehealth patient ratio is one of the most important operating cost factors”, and proposes $27/ month for a program that employs one full-time telehealth nurse per 200 patients [11]. The literature search yielded the willingness-to-pay for telehealth services in general [12–15], and the calculations were based on the following input: a one-off annual charge of $50 plus monthly subscription of $25 USD ($275 per year, for 11 months) for the service. The median salaries were reported per country on Payscale (www.payscale.com) and converted to US dollars. Table 1 lists all the inputs to the financial analysis.

**Table 1:** Inputs to the financial assessment of the B2C telemonitoring of chronic heart failure (in US dollars)

Items	Inputs
Sign-up/maintenance fee (per year)	$50
Subscription fee (per month)	$25
Support technician (IT) remuneration in Singapore (per year)	$21,162
Support technician (IT) remuneration in The Netherlands (per year)	$26,999
Support technician (IT) remuneration in the USA (per year)	$41,606
Registered nurse remuneration in Singapore (per year)	$26,889
Registered nurse remuneration in The Netherlands (per year)	$24,102
Registered nurse remuneration in the USA (per year)	$58,371
Operation manger remuneration in Singapore (per year)	$48,629
Operation manger remuneration in The Netherlands (per year)	$58,200
Operation manger remuneration in the USA (per year)	$60,572
Office rent (per employee, per month)	$600
Office supplies (per employee, per year)	$200
Call-centre services (per agent, per month)	$50
Back-end services (per month)	$189
App development (per year)	$750,000
Video education (52 videos per year)	$250,000
Mass-media promotion (per addressable market member)	$1.00
Cost to Acquire a Loyal User (CALU)	$2.51

*Remuneration consisting of salary, bonus, profit sharing and commission (Source:* *www.payscale.com* *)*

*Cost per Loyal User Index: April 2016 (Source* *www.fiksu.com* *)*

## Results

The business model assessment of a fictitious venture for telemonitoring patients with CHF is based on the following categories: venture type, six-factor alignment, strategic market assessment, financial viability, valuation analysis, sustainability, societal impact, and technology assessment. The caveats will be considered in the discussion.

### Venture type

The B2C model shifts the focus in telemonitoring away from technology (i.e. product focus) to the consumer (i.e. service focus). The plan is to build the venture on the foundations of an established telemonitoring business by extending the B2B model towards the B2C. Although the model uses a strong technology base, i.e. the back-end with patient stratification and data analysis systems, the focus is clearly on engaging patients and involving them directly in their care via measurements, education and targeted communication. The distribution channels for the service provision are digital - mobile communication networks with internet access – and the point-of-care are smartphones. Thus, the distribution channels are curtailing hospitals, and the service can be run on a regional or national level. These are all hallmarks of a *consumer-facing* venture.

### Six-factor alignment

**Fig. 3:**
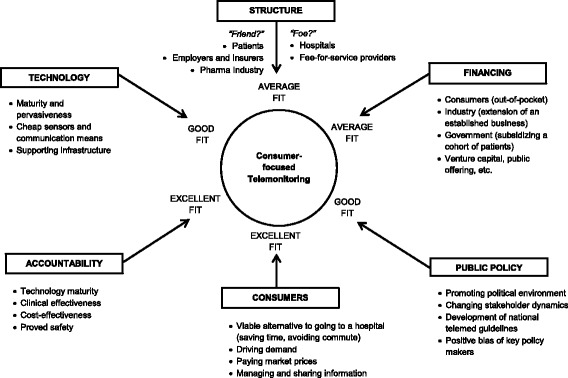
Six factors relevant to the B2C telemonitoring service for chronic heart failure patients. The Six factors analysis shows the relevant factors for implementation of B2C telemonitoring and the driving forces behind each factor. The list is not exhaustive. *Adapted from HBS Case No. 313–070, Rev. 2014 (Boston: Harvard Business School Publishing, 2014*

The analysis of the six factors critical to a B2C telemonitoring service for CHF patients is presented in Fig. 3.

#### Structure: “Average”

There are potential “friends” and “foes” in the contemporary healthcare system, looking to help or attack the innovator [4, 16]. The structural components for a telemonitoring venture are already present, but some are part of the healthcare organizations willing to preserve the *status quo*. The “friends” of this venture are the patients, and additional parties affected by a patient’s worsened or prevented heart failure exacerbation, including employers and insurance companies, as well as drug manufacturers given sub-optimal levels of medication adherence for which closer management may lead to increased use. The “foes” would be the hospitals and the healthcare systems operating in the fee-for-service mode, that includes conventional management of outpatient care in which patients show up for billable appointments (not at a hospital); however, with improved care, no billable service would be needed. The structure for the B2C telemonitoring is deemed *average* – all parts are in existence but need a skilful rearrangement so that all stakeholders remain “on board” (especially the physicians).

#### Financing: “Average”

B2C telemonitoring allows patients to compare and contrast the service offering with other market alternatives, as they pay “out of their own pocket”. This guarantees the market price in a given jurisdiction over time. In contrast, in the B2B model the insurance pays hospitals for the telemonitoring service and the equipment physicians and nurses use. The telemonitoring equipment is given to patients by the providers, and taken back after a certain period of time. The B2C model therefore removes the true bottleneck in implementation of telemonitoring – reimbursement [17]. The financing of a B2C telemonitoring venture does not rule out other sources of capital – e.g. venture capital, government subsidies, public offering, and loans.

#### Public policy alignment: “Good”

The following four variables influence public policy: political environment, stakeholder dynamics, regulatory and legislative process dynamics, and biases of key policy makers [4]. The political environment can help promote B2C telemonitoring as it takes the pressure off governments to provide healthcare services. It also reduces the need to train more healthcare staff to care for the rising number of patients with chronic diseases. This can, however, disrupt the current stakeholder dynamics, with some physicians viewing automatic telemonitoring as a threat to their jobs. B2C telemonitoring allows less expensive workers, i.e. telenurses, to take over the mundane part of the care process, while physicians remain in charge of a patient’s therapy. Regulatory dynamics can change over time, but for the time being national telemedicine guidelines have been developed, e.g. in Singapore [18]. In 2004 the United States administration established a dedicated office to promote the use of information technology in healthcare, allowing all Americans to have access to electronic health information [19]. This has resulted in a dramatic increase in funding for health IT, mainly spent on EMR systems, but it could also help the implementation of telemonitoring.

#### Technology alignment: “Good”

Technology innovators face two main problems: knowing when to invest in the technology, and who the competitors are [4]. Early telemonitoring solutions did not catch on, because technology was not mature enough and the healthcare “ecosystem” was not ready. But today the information-communication channels are available, the mobile devices are pervasive, and the push toward consumer-driven healthcare is real. Technology solutions compete not only with each other but also with other services and solutions in the healthcare ecosystem. Telemonitoring CHF can, and should, reduce the consumption of drugs, making the pharma industry a contender. Competition can also occur between outpatient clinics which used to manage chronic disease patients and the telemonitoring venture. Another source of conflict is data privacy and data ownership. ICT companies, governments, patients’ protection organizations all compete for data ownership, and have conflicting agendas. But as we assess the timing and the ecosystem for implementation of B2C telemonitoring, we believe that today there is a *good* alignment.

#### Consumer alignment: “Excellent”

There are two important trends in healthcare: empowerment, and lack of leisure time [4]. Patients now use the internet to search for information about their disease [20]. In Singapore and the Netherlands 25% and 21% respectively of the daily mobile internet users older than 55 years purchased a service or a product via smartphone [21]. Consumers of healthcare services in the United States spent $34 billion dollars of their own money on complementary and alternative medicine (CAM) in 2007, while making 354.2 million visits to practitioners of CAM [22]. Consumers might recognize telemonitoring as a viable way of managing their condition, besides going to a physician [23]. Statistics from The World Bank [24] show that in 2013 63% of the population in the United States, 64% in the Netherlands, and 68% in Singapore was in work. Statistics from OECD [25] show that on average per person each year 1788 h were worked in the United States and 1421 in the Netherlands (Singapore was not included). This data shows that leisure time is squeezed and that patients can benefit from not spending time going to see a physician or sitting in a waiting room. The B2C model helps patients engage with a healthcare system on their own terms, when and where they want, gaining “peace of mind” along the way, and thus the consumer alignment is deemed *excellent*.

#### Accountability: “Excellent”

Nowadays telemonitoring is safe and reliable [23] thanks to sensor and communication maturity. The research and development needed for a B2C telemonitoring service to deliver on its promise lies in the domain of ICT, patent protection and legal frameworks. The accountability toward the stakeholders is assured as several studies have proven that telemonitoring is: 1) effective, helping the industry with the implementation of the early systems [26, 27], 2) safe, helping the healthcare providers to accept the new ways of working [28, 29], and 3) cost-effective, helping governments/insurers with reimbursement for the new technology [30, 31]. Overall, the accountability of the B2C telemonitoring can be evaluated as *excellent.*

### Strategic market assessment

There is limited evidence of the prevalence of CHF in Asia, but the range is 1.26–6.7% [32]. We used the lower bound of 1.25%, and in Singapore that amounts to 62,500 patients in a population of approximately 5 million. With a smartphone penetration of 88% in the population [33], the highest in the world, Singapore is the obvious choice for a B2C telemonitoring pilot. In the age group 55+ some 62% of people have a smartphone, so most CHF patients are potential users of telemonitoring services.

Assuming the same estimated prevalence of CHF as in the general population – the market in the Netherlands represents 1.25% of an estimated population of 17 million – i.e. some 212,500 people. The Consumer Barometer and The Connected Consumer Survey 2014/2015 [34] show that 50% of the 55+ age group in the Netherlands use a smartphone. Again, with the rise in smartphone penetration in this age segment it is to be expected that the majority of potential CHF patients can be considered potential users of the telemonitoring service.

The same survey [33] shows that in the United States the smartphone penetration in the age group 55+ is 28%, while 71% use either a smartphone or a basic mobile phone. The CHF prevalence of 1.25% in approximately 320 million citizens amounts to 4 million people. The market opportunity in the United States is evidently big, but the challenge to provide the service is equally big, as the population is geographically dispersed and not homogeneous.

### Financial viability

A literature search yielded the willingness-to-pay for telemonitoring services. American Well conducted a survey in telehealth in the United States, and found that 64% of people would see a physician over video, but 62% think it should cost less than the current $82 for first-time patients [12]. Qureshi et al. found that “the majority of those choosing telemedicine (95%) were also willing to pay a median of $25 (5 to 500 dollars) out-of-pocket” [14]. Bradford et al. [13] found that 30–50% of hypertensive patients were willing to pay at least $20 per month (CHF patients were willing to pay even more), while Seto [15] found that 55% of heart-failure patients were willing to pay $20, and 19% were willing to pay $40.

#### Breakeven analysis

The breakeven volume of customers (i.e. CHF patients) in Singapore is 9877 and in The Netherlands it is 9451. That is 15.8% of the total CHF population in Singapore, and 4.45% in The Netherlands (based on 1.25% prevalence rate). The service is not viable in the United States due to scenario inputs (i.e. high median salaries), and does not break even. The total expenses, consisting of personnel and non-personnel expenses, are not offset by the fees collected in the United States.

#### Market share analysis

In the period from 1st June 2014 to 31st May 2015 there were 4085 admissions due to Heart Failure in Singapore (all hospitals and all wards combined), with each admission costing around $1500 SGD in Ward C (app. $1100 USD) and around $6000 SGD in Wards B1 and A (app. $4400 USD) [35]. As Singapore ages, these figures are likely to increase. Given that the total number of CHF patients in Singapore is estimated at 62,500, with 4085 (6.54%) being hospitalized, and the number of patients needed to break even is 9877 (15.8%), this venture seems viable and able to grow the market share. The calculations for the Netherlands are even more favourable due to the low percentage of patients needed to break even. However, due to the increased complexity of the service provision, factors should be applied to calculations in order to address the scalability and transferability issues (logistics, legal issues, care coordination, recruitment and training of nurses, customer acquisition, and media coverage).

### Valuation analysis

Here we present the valuation analysis (i.e. terminal value plus annual cash flows), to help us understand the business valuation after five years in Singapore, the Netherlands and the United States. The calculations assume a market share of 100% in the first two years (dropping to 55% in the sixth year) due to the “first mover advantage”, the competitive strategy used. The same percentage in a proportionately bigger country is proportionately harder to attain. However, for the comparability of the analyses all calculations assume a fixed market share per year and are presented at the same three levels of expected return on funds invested: 50%, 25% and 15% per year (Table 2). See Additional file 2 for detailed calculations.

**Table 2:** Valuation of the B2C telemonitoring venture in the fifth year (in million US dollars)

Country	Discount	Annual cash flow (profit/loss)	Present value of annual cash flow	Present value of all annual cash flows	Present value of terminal value^a^	Total present value^b^
Singapore	50% annually	3.13	0.41	1.51	4.13	5.64
	25% annually	3.13	1.03	3.15	10.27	13.41
	15% annually	3.13	1.56	4.42	15.58	20.00
Netherlands	50% annually	10.76	1.42	6.06	14.17	20.23
	25% annually	10.76	3.53	12.12	35.27	47.39
	15% annually	10.76	5.35	16.80	53.51	70.31
USA	50% annually	−94.76	0	0	0	0
	25% annually	−94.76	0	0	0	0
	15% annually	−94.76	0	0	0	0

^a^ *Terminal value calculated as ×10 annual cash flow in the fifth year*

^b^ *Sums not matching due to rounding*

From Table 2 we see that the B2C telemonitoring model is not only viable but also valuable. Given that the Singapore population is relatively small (roughly 5 million) and the target disease is of low prevalence (1.25%), the B2C model allows for a financially healthy venture valued at around $20 million US dollars (at 15% return annually), with around $3 million USD cash flow in the fifth year. The valuation of a similar venture in The Netherlands, which is a medium-sized country in terms of population (roughly 17 million) with the same disease prevalence (1.25%), comes to more than $70 million US dollars (at 15% return annually) with an annual cash flow of more than $10 million USD in the fifth year. In the United States, which has the largest population of the three (roughly 320 million), the same disease prevalence and the same non-personnel input cost, the venture is not viable.

### Sustainability

Furthermore, we explore the sustainability of the B2C telemonitoring from four perspectives: revenues, costs, management, and technology.

#### Revenue sustainability

The lifetime customer value of a CHF patients is low, due to the nature of the illness and its progression. In the case of a regular churn – the median survival after one year in CHF patients in the Framingham study was 57% for men and 64% for women [36] – a feasible customer acquisition strategy would aim at creating critical mass to ensure breakeven volumes and generate profit. Demand for these types of services, and for telehealth in general, will likely increase in the future as the population ages [37], adding to revenue sustainability.

#### Cost sustainability

In the B2C venture the biggest cost contribution is from the nurses’ salaries, followed by the salaries of the technicians and the managers. The non-personnel costs of the digital services will remain the same or drop in the future. The marginal cost of acquiring a new user might not equal zero, as this cost is a function of marketing and sales operations, but the cost of adding a new user to the system is obviously sufficiently low to be assumed zero [38]. Furthermore, we believe the telemonitoring equipment (i.e. sensors) will become commoditized in the future and costs will only be incurred for running the service (the B2C model in telemonitoring is device-agnostic). Frost & Sullivan [39] predict a decrease in telemonitoring equipment prices and an increase in telemonitoring service fees, with increased demand for telemonitoring via telehealth or telecare services in the future. The costs, from the provider perspective, will thus be tied to service management and not device manufacture.

#### Management sustainability

The management of chronic diseases is increasingly being seen as the job of registered nurses (RNs). In telemonitoring “the most frequent activity by the nurses was reporting information to the primary care provider (17%), followed by providing lifestyle information to the patient related to diabetes mellitus and hypertension (e.g., diet, smoking cessation, foot care [14%], and social contact with the patient [14%])” [40]. At the beginning of the century, it was expected that the shortage of nurses in the United States would continue until 2020 [41]. The employment projections for 2012–2022 released by the Bureau of Labour Statistic [42] in December 2013 confirm this, and the RN workforce is expected to grow from 2.71 million in 2012 to 3.24 million in 2022. This shortage in supply of (tele)nurses will reflect negatively on the scalability of this telemonitoring venture in the short run.

#### Technology sustainability

The main factor affecting sustainable provision of the service is not the telemonitoring technology but the inability to make the shift from a myriad of small-scale pilots to large-scale deployment, and the integration with contemporary healthcare systems [43]. In the near future, the smartphone manufacturers (or telecom operators) might initiate their own monitoring service. The production of devices and sensors might be commoditized, pushing the device manufacturers toward the service business. The sustainability of the technology is tied to successful implementation in the healthcare system, which in turn will feed another development cycle. This indeed is a “virtuous cycle” between technology development and service provision.

### Societal impact

Telemonitoring solutions for heart failure help patients to better self-manage their condition, and provide peace of mind for both patients and caregivers [44]. Because the B2C model relies on payments from service users (i.e. patients), while the public healthcare sector derives the benefit, in terms of prevented hospitalizations and ER visits, this venture has a positive impact on society. It allows patients the freedom to consume healthcare services when and where they want, governments to rationalize common funds and facilities, industry to generate profits via business-model innovation, and telecoms to extend operations into healthcare. The combination of the B2C and the B2B model can be further extended to B2G as governments might subsidize a cohort of their most severe CHF patients. Thus, this is a truly “*do good – do well”* venture – it does good for patients and society and performs well for shareholders.

### Technological risk assessment

The technology chain consists of: 1) various sensors and devices that connect to a smartphone via Bluetooth connection, 2) a smartphone connected to the internet via Wi-Fi or mobile data, and 3) a back-end consisting of servers hosting patient data and various types of telemonitoring software. We expect sensors to become cheap, or even commoditized, smartphones to become ubiquitous, and services via internet to become available to most patients in the future. Table 3 lists issues and gives a risk assessment for B2C telemonitoring.

**Table 3:** Checklist for evaluating new healthcare technologies in the B2C telemonitoring of chronic heart failure

Issues	Assessment^a^	Explanation
1. Understanding the Black Box	Small risk	Telemonitoring (i.e. measuring and transmitting physiological signals) consists of two parts – the algorithms aiding the nurses in reviewing the physiological data coming from the patients, and the telecommunication technologies – both with widely understood scientific mechanisms.
2. Depth of research	Medium risk	There is a substantial amount of research on the clinical effectiveness of heart failure monitoring, with promising results, but not so much on cost-effectiveness. The available meta analyses show improved survival and better outcomes with telemonitoring, at same or higher costs.
3. Downside risks	Small risk	Telemonitoring does not interfere with bodily functions. The care is provided by registered telemonitoring nurses while cure is administered by physicians and pharmacists.
4. Financial considerations: • *Market acceptability to medical personnel?*	Medium risk	Telemonitoring technology is not excessively innovative or disruptive to the healthcare process. The business model (B2C) is an extension of the existing one (B2B), and the novelty revolves about logistics and operations.
• *Are technologies financially beneficial to adopters?*	Small risk	A chronically ill person will spend approximately 1–2% of their average monthly income on a telemonitoring service. There is no risk in “over-spending” and no financial risk for the patient but the payment-borne-by-consumers model demands high attractiveness of the service to customers.
• *Creation of “turf warfare” among different physician specialties?*	Medium risk	As B2C telemonitoring is directed toward the patients/consumers it is opening/creating a market and not encroaching on existing “turf”. However, telemonitoring centres can be seen by hospitals as competitors rather than complementary organizations.
• *Requirement for new types of medical personnel?*	Medium risk	There is risk associated with the creation of a telemonitoring centre staffed by telemonitoring nurses in any jurisdiction. Creating the site, drafting and training personnel is risky.
• *Do technologies fit existing coverage, coding, and payment regulations?*	Medium risk	With such a large population of heart-failure patients in the world today, the regulation is slowly turning to full coverage and payment for telemonitoring.
• *Do technologies create a product and/or customer pipeline?*	Small risk	More advanced monitoring systems and packages tailored to individual patients (or other chronic patients) can be introduced later by adding new customer “pipelines”.
• *Market size and ease of penetration*	Small risk	The market is not very large in Singapore and is therefore easier to penetrate. The US or EU markets are bigger but harder to penetrate. In terms of disease prevalence, the global market for telemonitoring CHF is similar to the general population, i.e. 1–2%.
5. Regulatory issues: Seriousness of Problem	High risk	The FDA in the US has started to look over the medical app market and it is likely that clearance will be needed (likely other jurisdictions will require regulatory oversight). At the moment this risk can be averted by carefully making associations with existing healthcare organizations.
6. Potential competition from other technologies	High risk	There’s not much protection in telemonitoring apart from the algorithmic core, resulting in fierce market competition if new players enter the field. However, a telemonitoring centre is a huge deterrent to any party wanting to go with a purely device/service-based business model.
7. Likelihood of obtaining a patent	Small risk	Patents have been granted on the technologies involved in telemonitoring, on algorithms and software. The business processes and the business model cannot be patented.
8. Production considerations	Small risk	The development of the software/app will have to follow regulatory guidelines. However, once in place and clear of production issues, the service can be easily upgraded and distributed.

*Adapted from HBS Case No. 313–070, Rev. 2014 (Boston: Harvard Business School Publishing, 2014)*

^a^ *Assessment scale: small risk, medium risk, high risk*

## Discussion

The objective of this paper was to assess the potential societal and corporate value of B2C telemonitoring for CHF patients. In doing the analyses we used the Innovating in Healthcare framework as described under Methods, and the Results were presented according to the elements of the framework. The six-factor fit was found to be *average* for Structure and Financing, *good* for Public Policy and Technology, and *excellent* for Consumers and Accountability (Fig. 3). The venture created on the basis of the B2C model is viable and profitable for the chosen parameters, in Singapore and The Netherlands, but not in the United States (Table 2).

The healthcare providers – physicians, nurses and pharmacists – are part of the existing healthcare *structures* that are burdened with waste and inefficiency [45]. Patients go to see their physicians when they can, instead of when they should. In the United States, in 2012, 82.1% of adults contacted a healthcare professional – totalling 1.2 billion visits to physician practices, hospital outpatient and emergency departments [46]. Some of these visits can be prevented with B2C telemonitoring. The analysis revealed that “friends” of this venture would be patients and informal caregivers, employers and insurance companies, as well as drug manufacturers. The “foes” would be the hospitals and the healthcare systems that still operate in the fee-for-service mode.

Weinhold, Gastaldi, and Häckl [43] ascertain that “telemonitoring is one of the most promising concepts in enabling patients’ self-management, relocating medical services and improving equity in access to high-quality care”. However, the same authors say the major problem with telemonitoring today is not the *technology*, but the inability to move from small-scale pilots to population-wide deployment. This is due to the restrictive business model of the implementation where hospitals are in charge of the service organization and provision. Extending the B2B model in telehealth to B2C model solves for this bottleneck.

In the EU the predominant way of providing telehealth services is via third parties – home-healthcare agencies or specialized hospitals – in essence the B2B model [47]. The B2C model envisions telemonitoring centres as separate entities, which host technology and nurses, and provide the service in a whole jurisdiction. Via this model, *consumers* are in charge of the service they consume, the data they produce, and the information they share. Consumer-driven healthcare is expected to become the norm in the twenty-first century, but there are problems, as shown in The Netherlands and Switzerland [48].

The business model is one of the impediments to wider adaptation of telemonitoring, while *financing* is another. In the healthcare sector revenues and capital are acquired in a different way than in most other industries [4]. In the developed world the reimbursement in healthcare usually goes via a third party – insurers or government – where the user does not pay, and the payer does not use the good or service [4]. The patients know very little about the cost of the treatment, and the prices can be determined by providers in a “non-market” way. With B2C telemonitoring the patients are aware of prices and can control costs.

The healthcare sector is heavily regulated by the government, as the government is the biggest purchaser [4]. From the government perspective, there is more incentive to protect and overregulate than to face public outcry if a drug or a treatment proves to be harmful [4]. The B2C telemonitoring needs all four stakeholders – creators, providers, distributors, and payers – to work together in order to succeed in a jurisdiction. This is a complex problem, although *public policy* favours telehealth as it saves costs and improves health-related outcomes [49].

Understanding the innovation type, the market size, the competitive strategy and the valuation of a venture is not enough. In addition to all of this, the impact of the venture on society should be positive. B2C telemonitoring is an example where all stakeholders are *accountable* and benefit from innovation – customers benefit from the service when they need it, healthcare benefits from the reduced burden and improved effectiveness, industry benefits from the creation of innovative businesses, and governments benefit from reduced expenditure while citizens enjoy the best possible care.

Thus, extending the B2B model toward B2C could be the next step in health services integration [50] because the B2C model in telemonitoring CHF patients supports:

1. Clinical Integration: the nurse makes sure that the cardiologist only gets involved in complex cases. The patient communicates with the nurse who makes sure others are informed about the status of the patient and interventions, if necessary;
2. Service Integration: the patient receives care at home and does not meet the nurse or the cardiologist at the hospital;
3. Financial Integration: the B2C model applies a monthly rate instead of the current fee-for-service payment structure. As such, it support financial integration. However, for true financial integration additional changes within the system are required.

Kannampallil et al. [51] proposed a theoretical lens for studying complexity in healthcare based on the *degrees of interrelatedness* of system components. Functional decomposition is proposed as a mechanism for studying the subcomponents and their interrelatedness. In the B2C model (Figs. 1 and 2) data, voice, money and drugs are continuously exchanged. In this framework we would position the B2C model in the NW part of the graph: *low number of components*, but *high degree of interrelatedness*.

### Limitations

The main limitation of our study is that we used a non-peer-reviewed method [4], and did not investigate the validity of the six-factor qualification fit. Notwithstanding the Framework not being subject to peer-review in the scientific literature, it was found in this study that the framework is easily applicable in the field of telehealth. Another major concern is the non-systematic search of the literature, and opting for a convenience sample. In the financial analysis we assumed that an equal percentage of market share can be achieved in all three geographies, that the churn of patients on the service will not be extreme due to death or withdrawal, and that patients will be able to spend approximately 1–2% of their net monthly income on the service. The analysis excluded the cost of the telemonitoring devices that the patients would need to procure. The efficiency estimates were taken over from literature and an interview with one telehealth provider (Hull, UK), while the cost of non-personnel expenses was arbitrary. We performed a scenario analysis, without a sensitivity analysis on the level of individual variables. Also, the same group of authors that created the model took part in the assessment, which might have introduced bias.

## Conclusion

The business model assessment revealed that B2C telemonitoring creates value for customers (patients), shareholders of the service provider, and society. The analysis of the healthcare ecosystem where this innovation could be implemented – Singapore, The Netherlands, or the United States – shows potentially a good-to-excellent fit of the model with the Six Factors. The financial analysis indicates that the venture is profitable, except in the United States, according to the chosen input parameters.

## Additional files

Additional file 1: B2C Model Assessment for Telemonitoring CHF. Innovating in Healthcare Framework by Herzlinger (2013). Description of data: List and overview of the framework items. (DOCX 16 kb)

Additional file 2: B2C Model Assessment for Telemonitoring CHF. Calculation inputs and Venture valuation calculations. Description of data: Tables listing cost inputs to MS Excel model, and venture valuation calculations, for Singapore, the Netherlands, and the United States. (DOCX 60 kb)

## Abbreviations

B2B: Business-to-Business
B2C: Business-to-Consumer
B2G: Business-to-Government
CAM: Complementary and Alternative Medicine
CHF: Chronic Heart Failure
ICT: Information and Communication Technology
RN: Registered Nurse
USD: United States Dollars
