# Supplementary material for: Assessment of a Business-to-Consumer (B2C) model for Telemonitoring patients with Chronic Heart Failure (CHF)
Source: BMC Med Inform Decis Mak. 2017 Oct 11;17:145. doi: 10.1186/s12911-017-0541-2 (PMC5637089; doi:10.1186/s12911-017-0541-2)
Supplement: Supplementary file 1 — B2C Model Assessment for Telemonitoring CHF. Innovating in Healthcare Framework by Herzlinger (2013). Description of data: List and overview of the framework items. (DOCX 16 kb) [file 12911_2017_541_MOESM1_ESM.docx]

# ADDITIONAL FILE 1 – Innovating in Healthcare Framework by Herzlinger (2013)

Caveats - “Life is not literature” (reality is much more complex than the business plan), “Basic is beautiful” (real-world numbers need to support the analysis), and “Beware of true believers” (zealots devoted to the proposed product and services bias the analysis).

1. **The Three Distinctly Different Types of Healthcare Innovation**
2. Consumer-focused Ventures

These innovative healthcare ventures increase productivity in two ways:

1. Consumer-focused innovations involve the consumer in the healthcare services delivery process and thus reduce healthcare expenditure and enhance quality.
2. Consumer-focused innovations increase productivity by reducing the time the patient spends waiting, coordinating, and travelling – for example “focused factories” that integrate the healthcare delivery process from the patients’ perspective and are consciously systematic about designing the process for the care they offer.
3. Technology-based Ventures

Diagnostics, medical devices, drugs and drug-delivery systems, are examples of the technology that enables a higher quality of care.

1. Integrator Ventures

Innovative integrator healthcare ventures bring the benefits of economies of scale to the fragmented care structure, which is lacking in efficiency and standardization, in two ways:

1. They vertically integrate many diverse providers into one seamless system of care, for example through integrated health service delivery networks and technology firms that provide one-stop shopping for their customers (e.g. Amil, a Brazilian health insurer)
2. They horizontally integrate fragmented, lone-ranger healthcare providers to provide better treatment at lower costs (e.g. the Hospital Corporation of America’s integration of 150 hospitals into one chain).
3. **Six-factor Alignment: Is the Idea Viable?**
4. Structure

There are many friends and foes of new ventures lurking in the system, each with substantial resources and power to attack or help the innovator.

1. Financing

The process for obtaining revenues (e.g. reimbursement) and acquiring capital in the healthcare sector differ from those in most other industries.

1. Public Policy

Because of the irreversible, costly damage that incompetent or fraudulent suppliers can cause and because governments are major purchasers of healthcare, the sector is heavily regulated by many different authorities.

1. Technology

Technology innovators face two key managerial questions:

1. When to invest in the technology?
2. Who are my competitors?
3. Consumers

Consumers feel strongly about healthcare and influence it in many ways. Two key characteristics underlying these consumer activities in healthcare are:

1. Empowerment
2. Lack of leisure time
3. Accountability

Increasingly, empowered consumers and cost-pressured payers demand accountability from healthcare innovators.

1. **Business Model Elements: How to Make It Happen**
2. Strategic Market Assessment
3. Obtaining Market Information (Sizing the Market)
4. Converting Research Findings into a Marketing Plane
5. Competitive Strategy (First Movers, Fast Followers, Legal Barriers)
6. Financial Viability
7. Breakeven Volume
8. Market Share Analysis
9. Valuation Analysis
10. Cash Flows
11. Required Rates of Return
12. Terminal Value
13. Exit Value Analysis
14. Sustainability
15. Revenues
16. Costs
17. Management
18. Technology
19. Managerial Assessment
20. Fit of Managerial Skills and Opportunity
21. “Wannabes” versus “Experienced” Managers
22. Societal Impact
23. Technological Risk Assessments
24. Understanding the “Black Box”
25. Depth of research
26. Downside Risks
27. Financial Considerations
28. Regulatory Issues
29. Potential Competition from Other technologies
30. Likelihood of a Patent
31. Production Considerations
