# Supplementary material for: Assessment of a Business-to-Consumer (B2C) model for Telemonitoring patients with Chronic Heart Failure (CHF)
Source: BMC Med Inform Decis Mak. 2017 Oct 11;17:145. doi: 10.1186/s12911-017-0541-2 (PMC5637089; doi:10.1186/s12911-017-0541-2)
Supplement: Supplementary file 2 — B2C Model Assessment for Telemonitoring CHF. Calculation inputs and Venture valuation calculations. Description of data: Tables listing cost inputs to MS Excel model, and venture valuation calculations, for Singapore, the Netherlands, and the United States. (DOCX 60 kb) [file 12911_2017_541_MOESM2_ESM.docx]

## Table 1 – Calculation inputs for Singapore (in US dollars)

| **Year** | **2017** | **2018** | **2019** | **2020** | **2021** | **2022** |
| --- | --- | --- | --- | --- | --- | --- |
| Technology Penetration | 70.4% | 74.9% | 78.9% | 82.3% | 85.4% | 88.3% |
|  |  |  |  |  |  |  |
| Addressable Market | 43,996 | 46,835 | 49,294 | 51,464 | 53,404 | 55,160 |
| Market Adoption | 2.5% | 16.0% | 50.0% | 84.0% | 97.5% | 100.0% |
| Total Market | 1,100 | 7,494 | 24,647 | 43,230 | 52,069 | 55,160 |
| Market Share Projection | 100.0% | 100.0% | 95.0% | 85.0% | 70.0% | 55.0% |
|  |  |  |  |  |  |  |
| Patients Enrolled | 1,100 | 7,494 | 23,415 | 36,745 | 36,449 | 30,338 |
| Technicians Needed | 1 | 3 | 9 | 15 | 14 | 12 |
| Nurses Needed | 7 | 44 | 135 | 212 | 210 | 175 |
| Managers Needed | 1 | 2 | 5 | 8 | 8 | 7 |
|  |  |  |  |  |  |  |
| Personnel Costs (Technicians) | $21,162 | $63,487 | $190,461 | $317,434 | $296,272 | $253,948 |
| Personnel Costs (Nurses) | $188,220 | $1,183,096 | $3,629,954 | $5,700,373 | $5,646,596 | $4,705,496 |
| Personnel Costs (Managers) | $48,629 | $97,258 | $243,145 | $389,032 | $389,032 | $340,403 |
|  |  |  |  |  |  |  |
| Office Rent | $21,600 | $117,600 | $357,600 | $564,000 | $556,800 | $465,600 |
| Office Supplies | $1,800 | $9,800 | $29,800 | $47,000 | $46,400 | $38,800 |
| Call-center Services | $4,800 | $28,200 | $86,400 | $136,200 | $134,400 | $112,200 |
| Back-end Services | $2,495 | $16,995 | $53,105 | $83,338 | $82,665 | $68,807 |
| App Development | $750,000 | $750,000 | $750,000 | $750,000 | $750,000 | $750,000 |
| Video Education | $250,000 | $250,000 | $250,000 | $250,000 | $250,000 | $250,000 |
| Promotion | $43,996 | $46,835 | $49,294 | $51,464 | $53,404 | $55,160 |
| Customer Acquisition | $2,761 | $18,809 | $58,771 | $92,230 | $91,486 | $76,148 |
|  |  |  |  |  |  |  |
| Expenses (no overhead) | $1,335,462 | $2,582,081 | $5,698,531 | $8,381,072 | $8,297,056 | $7,116,562 |
| Overhead | $66,773 | $129,104 | $284,927 | $419,054 | $414,853 | $355,828 |
| Total Expenses | $1,402,235 | $2,711,185 | $5,983,458 | $8,800,126 | $8,711,909 | $7,472,390 |
|  |  |  |  |  |  |  |
| Revenue | $357,465 | $2,435,419 | $7,609,833 | $11,942,193 | $11,845,783 | $9,859,847 |
|  |  |  |  |  |  |  |
| Profit/Loss | -$1,044,770 | -$275,766 | $1,626,375 | $3,142,068 | $3,133,874 | $2,387,457 |
| Cumulative Profit/Loss | -$1,044,770 | -$1,320,536 | $305,840 | $3,447,907 | $6,581,782 | $8,969,238 |

## Table 2 – Venture valuation calculation for Singapore (in US dollars)

| **Year** | **1** | **2** | **3** | **4** | **5** | **6** |
| --- | --- | --- | --- | --- | --- | --- |
| **50% return on investment** |  |  |  |  |  |  |
| Annual Cash Flow (Profit/Loss) | -$1,044,770 | -$275,766 | $1,626,375 | $3,142,068 | $3,133,874 | $2,387,457 |
| Present Value of Annual Cash Flow | $0 | $0 | $481,889 | $620,655 | $412,691 | $209,598 |
| Present Value of All Annual Cash Flows | $0 | $0 | $481,889 | $1,102,544 | $1,515,236 | $1,724,834 |
| Present Value of Terminal Value (x10) | $0 | $0 | $4,818,890 | $6,206,553 | $4,126,913 | $2,095,984 |
| Valuation | $0 | $0 | $5,300,779 | $7,309,098 | $5,642,148 | $3,820,818 |
|  |  |  |  |  |  |  |
| **25% return on investment** |  |  |  |  |  |  |
| Annual Cash Flow (Profit/Loss) | -$1,044,770 | -$275,766 | $1,626,375 | $3,142,068 | $3,133,874 | $2,387,457 |
| Present Value of Annual Cash Flow | $0 | $0 | $832,704 | $1,286,991 | $1,026,908 | $625,857 |
| Present Value of All Annual Cash Flows | $0 | $0 | $832,704 | $2,119,695 | $3,146,603 | $3,772,460 |
| Present Value of Terminal Value (x10) | $0 | $0 | $8,327,041 | $12,869,909 | $10,269,080 | $6,258,574 |
| Valuation | $0 | $0 | $9,159,746 | $14,989,604 | $13,415,683 | $10,031,035 |
|  |  |  |  |  |  |  |
| **15% return on investment** |  |  |  |  |  |  |
| Annual Cash Flow (Profit/Loss) | -$1,044,770 | -$275,766 | $1,626,375 | $3,142,068 | $3,133,874 | $2,387,457 |
| Present Value of Annual Cash Flow | $0 | $0 | $1,069,368 | $1,796,487 | $1,558,089 | $1,032,163 |
| Present Value of All Annual Cash Flows | $0 | $0 | $1,069,368 | $2,865,856 | $4,423,945 | $5,456,108 |
| Present Value of Terminal Value (x10) | $0 | $0 | $10,693,682 | $17,964,874 | $15,580,895 | $10,321,634 |
| Valuation | $0 | $0 | $11,763,050 | $20,830,729 | $20,004,840 | $15,777,743 |

## Table 3 - Calculation inputs for the Netherlands (in US dollars)

| **Year** | **2017** | **2018** | **2019** | **2020** | **2021** | **2022** |
| --- | --- | --- | --- | --- | --- | --- |
| Technology Penetration | 53.0% | 56.3% | 59.0% | 61.5% | 63.7% | 65.7% |
|  |  |  |  |  |  |  |
| Addressable Market | 112,727 | 119,557 | 125,474 | 130,692 | 135,360 | 139,583 |
| Market Adoption | 2.5% | 16.0% | 50.0% | 84.0% | 97.5% | 100.0% |
| Total Market | 2,818 | 19,129 | 62,737 | 109,781 | 131,976 | 139,583 |
| Market Share Projection | 100.0% | 100.0% | 95.0% | 85.0% | 70.0% | 55.0% |
|  |  |  |  |  |  |  |
| Patients Enrolled | 2,818 | 19,129 | 59,600 | 93,314 | 92,383 | 76,771 |
| Technicians Needed | 2 | 8 | 23 | 36 | 36 | 30 |
| Nurses Needed | 17 | 110 | 343 | 537 | 532 | 442 |
| Managers Needed | 1 | 4 | 13 | 20 | 19 | 16 |
|  |  |  |  |  |  |  |
| Personnel Costs (Technicians) | $53,998 | $215,991 | $620,975 | $971,962 | $971,962 | $809,968 |
| Personnel Costs (Nurses) | $409,737 | $2,651,241 | $8,267,051 | $12,942,875 | $12,822,364 | $10,653,167 |
| Personnel Costs (Managers) | $58,200 | $232,799 | $756,595 | $1,163,993 | $1,105,793 | $931,194 |
|  |  |  |  |  |  |  |
| Office Rent | $48,000 | $292,800 | $909,600 | $1,423,200 | $1,408,800 | $1,171,200 |
| Office Supplies | $4,000 | $24,400 | $75,800 | $118,600 | $117,400 | $97,600 |
| Call-center Services | $11,400 | $70,800 | $219,600 | $343,800 | $340,800 | $283,200 |
| Back-end Services | $6,392 | $43,385 | $135,173 | $211,636 | $209,525 | $174,116 |
| App Development | $750,000 | $750,000 | $750,000 | $750,000 | $750,000 | $750,000 |
| Video Education | $250,000 | $250,000 | $250,000 | $250,000 | $250,000 | $250,000 |
| Promotion | $112,727 | $119,557 | $125,474 | $130,692 | $135,360 | $139,583 |
| Customer Acquisition | $7,074 | $48,014 | $149,596 | $234,218 | $231,882 | $192,694 |
|  |  |  |  |  |  |  |
| Expenses (no overhead) | $1,711,527 | $4,698,987 | $12,259,863 | $18,540,976 | $18,343,886 | $15,452,723 |
| Overhead | $85,576 | $234,949 | $612,993 | $927,049 | $917,194 | $772,636 |
| Total Expenses | $1,797,104 | $4,933,936 | $12,872,856 | $19,468,025 | $19,261,081 | $16,225,359 |
|  |  |  |  |  |  |  |
| Revenue | $915,910 | $6,216,976 | $19,369,973 | $30,327,086 | $30,024,576 | $24,950,461 |
|  |  |  |  |  |  |  |
| Profit/Loss | -$881,194 | $1,283,040 | $6,497,116 | $10,859,061 | $10,763,495 | $8,725,102 |
| Cumulative Profit/Loss | -$881,194 | $401,846 | $6,898,962 | $17,758,023 | $28,521,518 | $37,246,620 |

## Table 4 – Venture valuation calculation for the Netherlands (in US dollars)

| **Year** | **1** | **2** | **3** | **4** | **5** | **6** |
| --- | --- | --- | --- | --- | --- | --- |
| **50% return on investment** |  |  |  |  |  |  |
| Annual Cash Flow (Profit/Loss) | -$881,194 | $1,283,040 | $6,497,116 | $10,859,061 | $10,763,495 | $8,725,102 |
| Present Value of Annual Cash Flow | $0 | $570,240 | $1,925,071 | $2,145,000 | $1,417,415 | $765,990 |
| Present Value of All Annual Cash Flows | $0 | $570,240 | $2,495,311 | $4,640,311 | $6,057,726 | $6,823,716 |
| Present Value of Terminal Value (x10) | $0 | $5,702,398 | $19,250,715 | $21,449,997 | $14,174,150 | $7,659,898 |
| Valuation | $0 | $6,272,638 | $21,746,026 | $26,090,308 | $20,231,876 | $14,483,613 |
|  |  |  |  |  |  |  |
| **25% return on investment** |  |  |  |  |  |  |
| Annual Cash Flow (Profit/Loss) | -$881,194 | $1,283,040 | $6,497,116 | $10,859,061 | $10,763,495 | $8,725,102 |
| Present Value of Annual Cash Flow | $0 | $821,145 | $3,326,524 | $4,447,871 | $3,526,982 | $2,287,233 |
| Present Value of All Annual Cash Flows | $0 | $821,145 | $4,147,669 | $8,595,540 | $12,122,522 | $14,409,755 |
| Present Value of Terminal Value (x10) | $0 | $8,211,454 | $33,265,235 | $44,478,713 | $35,269,820 | $22,872,332 |
| Valuation | $0 | $9,032,599 | $37,412,904 | $53,074,253 | $47,392,342 | $37,282,087 |
|  |  |  |  |  |  |  |
| **15% return on investment** |  |  |  |  |  |  |
| Annual Cash Flow (Profit/Loss) | -$881,194 | $1,283,040 | $6,497,116 | $10,859,061 | $10,763,495 | $8,725,102 |
| Present Value of Annual Cash Flow | $0 | $970,162 | $4,271,959 | $6,208,703 | $5,351,359 | $3,772,102 |
| Present Value of All Annual Cash Flows | $0 | $970,162 | $5,242,122 | $11,450,825 | $16,802,184 | $20,574,287 |
| Present Value of Terminal Value (x10) | $0 | $9,701,623 | $42,719,594 | $62,087,033 | $53,513,593 | $37,721,024 |
| Valuation | $0 | $10,671,785 | $47,961,716 | $73,537,858 | $70,315,777 | $58,295,311 |

## Table 5 – Calculation inputs for the US (in US dollars)

| **Year** | **2017** | **2018** | **2019** | **2020** | **2021** | **2022** |
| --- | --- | --- | --- | --- | --- | --- |
| Technology Penetration | 36.1% | 37.4% | 38.5% | 39.5% | 40.4% | 41.2% |
|  |  |  |  |  |  |  |
| Addressable Market | 1,444,831 | 1,496,626 | 1,541,492 | 1,581,067 | 1,616,469 | 1,648,493 |
| Market Adoption | 2.5% | 16.0% | 50.0% | 84.0% | 97.5% | 100.0% |
| Total Market | 36,121 | 239,460 | 770,746 | 1,328,097 | 1,576,057 | 1,648,493 |
| Market Share Projection | 100.0% | 100.0% | 95.0% | 85.0% | 70.0% | 55.0% |
|  |  |  |  |  |  |  |
| Patients Enrolled | 36,121 | 239,460 | 732,209 | 1,128,882 | 1,103,240 | 906,671 |
| Technicians Needed | 14 | 92 | 281 | 433 | 423 | 348 |
| Nurses Needed | 208 | 1,377 | 4,211 | 6,492 | 6,344 | 5,214 |
| Managers Needed | 8 | 49 | 150 | 231 | 226 | 186 |
|  |  |  |  |  |  |  |
| Personnel Costs (Technicians) | $582,484 | $3,827,752 | $11,691,286 | $18,015,398 | $17,599,338 | $14,478,888 |
| Personnel Costs (Nurses) | $12,141,168 | $80,376,867 | $245,800,281 | $378,944,532 | $370,305,624 | $304,346,394 |
| Personnel Costs (Managers) | $484,576 | $2,968,028 | $9,085,800 | $13,992,132 | $13,689,272 | $11,266,392 |
|  |  |  |  |  |  |  |
| Office Rent | $552,000 | $3,643,200 | $11,140,800 | $17,174,400 | $16,783,200 | $13,795,200 |
| Office Supplies | $46,000 | $303,600 | $928,400 | $1,431,200 | $1,398,600 | $1,149,600 |
| Call-center Services | $133,200 | $881,400 | $2,695,200 | $4,155,000 | $4,060,200 | $3,337,200 |
| Back-end Services | $81,922 | $543,096 | $1,660,650 | $2,560,305 | $2,502,148 | $2,056,330 |
| App Development | $750,000 | $750,000 | $750,000 | $750,000 | $750,000 | $750,000 |
| Video Education | $250,000 | $250,000 | $250,000 | $250,000 | $250,000 | $250,000 |
| Promotion | $1,444,831 | $1,496,626 | $1,541,492 | $1,581,067 | $1,616,469 | $1,648,493 |
| Customer Acquisition | $90,663 | $601,045 | $1,837,844 | $2,833,494 | $2,769,132 | $2,275,744 |
|  |  |  |  |  |  |  |
| Expenses (no overhead) | $16,556,844 | $95,641,613 | $287,381,753 | $441,687,528 | $431,723,982 | $355,354,241 |
| Overhead | $827,842 | $4,782,081 | $14,369,088 | $22,084,376 | $21,586,199 | $17,767,712 |
| Total Expenses | $17,384,686 | $100,423,694 | $301,750,841 | $463,771,905 | $453,310,182 | $373,121,953 |
|  |  |  |  |  |  |  |
| Revenue | $11,739,253 | $77,824,542 | $237,967,883 | $366,886,704 | $358,552,939 | $294,668,090 |
|  |  |  |  |  |  |  |
| Profit/Loss | -$5,645,433 | -$22,599,152 | -$63,782,958 | -$96,885,201 | -$94,757,242 | -$78,453,863 |
| Cumulative Profit/Loss | -$5,645,433 | -$28,244,585 | -$92,027,543 | -$188,912,744 | -$283,669,987 | -$362,123,850 |

## Table 6 – Venture valuation calculation for the US (in US dollars)

| **Year** | **1** | **2** | **3** | **4** | **5** | **6** |
| --- | --- | --- | --- | --- | --- | --- |
| **50% return on investment** |  |  |  |  |  |  |
| Annual Cash Flow (Profit/Loss) | -$5,645,433 | -$22,599,152 | -$63,782,958 | -$96,885,201 | -$94,757,242 | -$78,453,863 |
| Present Value of Annual Cash Flow | $0 | $0 | $0 | $0 | $0 | $0 |
| Present Value of All Annual Cash Flows | $0 | $0 | $0 | $0 | $0 | $0 |
| Present Value of Terminal Value (x10) | $0 | $0 | $0 | $0 | $0 | $0 |
| Valuation | $0 | $0 | $0 | $0 | $0 | $0 |
|  |  |  |  |  |  |  |
| **25% return on investment** |  |  |  |  |  |  |
| Annual Cash Flow (Profit/Loss) | -$5,645,433 | -$22,599,152 | -$63,782,958 | -$96,885,201 | -$94,757,242 | -$78,453,863 |
| Present Value of Annual Cash Flow | $0 | $0 | $0 | $0 | $0 | $0 |
| Present Value of All Annual Cash Flows | $0 | $0 | $0 | $0 | $0 | $0 |
| Present Value of Terminal Value (x10) | $0 | $0 | $0 | $0 | $0 | $0 |
| Valuation | $0 | $0 | $0 | $0 | $0 | $0 |
|  |  |  |  |  |  |  |
| **15% return on investment** |  |  |  |  |  |  |
| Annual Cash Flow (Profit/Loss) | -$5,645,433 | -$22,599,152 | -$63,782,958 | -$96,885,201 | -$94,757,242 | -$78,453,863 |
| Present Value of Annual Cash Flow | $0 | $0 | $0 | $0 | $0 | $0 |
| Present Value of All Annual Cash Flows | $0 | $0 | $0 | $0 | $0 | $0 |
| Present Value of Terminal Value (x10) | $0 | $0 | $0 | $0 | $0 | $0 |
| Valuation | $0 | $0 | $0 | $0 | $0 | $0 |
